# Deletion and duplication of 16p11.2 are associated with opposing effects on visual evoked potential amplitude

**DOI:** 10.1186/s13229-016-0095-7

**Published:** 2016-06-27

**Authors:** Jocelyn J. LeBlanc, Charles A. Nelson

**Affiliations:** Division of Developmental Medicine, Laboratories of Cognitive Neuroscience, Boston Children’s Hospital, Boston, MA USA; Department of Neurology, F.M. Kirby Neurobiology Center, Boston Children’s Hospital, Boston, MA USA; Department of Neurobiology, Harvard Medical School, Boston, MA USA; Department of Pediatrics, Harvard Medical School, Boston, MA USA

**Keywords:** 16p11.2 copy number variation, Visual evoked potential, Visual cortex

## Abstract

**Background:**

Duplication and deletion of the chromosomal region 16p11.2 cause a broad range of impairments, including intellectual disability, language disorders, and sensory symptoms. However, it is unclear how changes in 16p11.2 dosage affect cortical circuitry during development. The aim of this study was to investigate whether the visual evoked potential (VEP) could be used as a noninvasive quantitative measure of cortical processing in children with 16p11.2 copy number variation.

**Methods:**

Pattern-reversal VEPs were successfully recorded in 19 deletion carriers, 9 duplication carriers, and 13 typically developing children between the ages of 3 and 14 years. The stimulus was a black and white checkerboard (60’) that reversed contrast at 2 Hz. VEP responses were extracted from continuous EEG recorded using a high-density elasticized electrode net.

**Results:**

Quantitative analysis of the VEP waveform revealed that, relative to controls, deletion carriers displayed increased amplitude and duplication carriers displayed diminished amplitude. Latencies of the VEP waveform components were unaffected by 16p11.2 status. P1 amplitude did not correlate with age, IQ, or head circumference.

**Conclusions:**

The results of this study suggest that recording VEP is a useful method to assay cortical processing in children with 16p11.2 copy number variation. There is a gene dosage-dependent effect on P1 amplitude that merits further investigation. The VEP is directly translatable to animal models, offering a promising way to probe the neurobiological mechanisms underlying cortical dysfunction in this developmental disorder.

## Background

Copy number variation of the 16p11.2 chromosomal region (16p CNV) is associated with developmental delay and a wide range of physical abnormalities, behavioral problems, and intellectual deficits [1]. Some features are similarly manifested in deletion and duplication carriers, such as seizures and cognitive impairment [1], and others are affected in opposite ways, including head circumference and body weight [2, 3]. This begs the question of whether brain function is affected in a gene dosage-dependent manner. Although progress is being made on characterizing the disorder and parsing the neurobiological mechanisms, there remains a gap in our understanding of how these genetic changes ultimately produce the varied cognitive and behavioral outcomes in this population.

Abnormal processing of sensory information is emerging as a common problem in neurodevelopmental disorders [4, 5], and testing sensory modalities like vision offers an easily accessible window into cortical processing. Abnormalities of basic sensory processing may contribute to and be indicative of global cortical deficits that would be expressed as intellectual and behavioral deficits. A recent study reported delayed auditory evoked responses using magnetoencephalography in deletion but not duplication carriers [5]. We were interested in whether this deficit would also pertain to the processing of visual information. Vision is an ideal system because it is evolutionarily conserved between rodents and humans, well characterized, easily tested in a standardized manner, and relevant to socialization, communication, and cognition.

Recording pattern-reversal visual evoked potentials (VEPs) is a powerful method to noninvasively assess the integrity of cortical processing in babies and children, even those with profound disabilities [4, 6]. Eliciting VEP involves a quick and passive procedure that only requires fixation on a monitor, and the evoked response is stable and robust enough to produce a recognizable waveform after only a few trials. The waveform shape elicited by this particular large check stimulus matures within the first 2 years of life [7–11], and group differences in latency and amplitude can be quantified [4, 12].

Here, we recorded pattern-reversal VEP in children carrying 16p deletions and duplications. Our goals were to assess if the VEP could detect differences in cortical processing between typically developing children and those with 16p CNV and between those with deletions and duplications. We predicted that the latency and/or amplitude of visual responses would either be similarly affected by 16p deletion and duplication due to a common effect of disruption of this chromosomal region on synapses and circuitry, or would be differentially affected by 16p copy number due to a gene dosage effect on the wiring of the visual cortex [5, 13, 14].

## Methods

### Participants

All procedures were reviewed and approved by the Boston Children’s Hospital (BCH) Office of Clinical Investigation and the BCH Institutional Review Board, and written informed consent was obtained from each participant or guardian prior to testing. Individuals carrying 16p11.2 deletions and duplications were recruited to participate in the study through the Simons Variation in Individuals Project (SVIP). Eligibility and exclusionary criteria are described previously [5]. Typically developing children were recruited through the BCH participant registry and did not have any neurological or developmental disorders, seizures, and ophthalmological problems. Data were collected onsite at BCH and at SVIP family meetings in Orlando, Florida, and Chicago, Illinois.

The final dataset included 19 deletion carriers and 12 duplication carriers and a comparison group of 15 typically developing controls between 3 and 14 years old. Several participants in the study were tested but were excluded from further VEP analysis because noncompliance prevented completion of data collection (2 duplication carriers), technical issues occurred at the time of acquisition (2 controls), or an insufficient number of trials remained after artifact detection (1 duplication carrier). Participant information is summarized in Table 1.

**Table 1:** Description of study participants

Group	*N*	Age (months) median, range	Gender	CNV inheritance			ASD		
				De novo	Inherited	?	Yes	No	?
Control	13	60 (39–165)	5 M	–	–	–	–	–	–
			8 F	–	–	–	–	–	–
Deletion	19	62 (43–163)	10 M	6	1	3	2	6	2
			9 F	7	1	1	3	6	0
Duplication	9	70 (40–122)	5 M	0	4	1	1	4	0
			4 F	1	2	1	0	3	1

*M* male, *F* female, *CNV* copy number variant, *ASD* autism spectrum disorder

Phenotypic information was obtained from the Simons Foundation Autism Research Initiative (SFARI) Base (www.sfari.org/resources/sfari-base). Full-scale intelligence quotient (IQ) scores were derived without adjusting for prematurity and were only used if taken within 6 months of the VEP session date. IQ was not assessed for typically developing participants because they were recruited independently through BCH and did not undergo the SFARI phenotypic battery. However, because the typically developing sample was drawn from a database of nearly 20,000 families and we have previously assessed IQ in a subset of these children as part of another project, we expect the IQ of our current group is average to above average. Autism spectrum disorder status was determined based on the diagnosis given by experienced, licensed clinicians. Seizure history was available for 11/19 deletion carriers and 6/9 duplication carriers through the SFARI neurologic record review form. Of these individuals, 4 deletion carriers and no duplication carriers had a history of seizures. Current medication information was available for 7/19 deletion carriers and 4/9 duplication carriers through the SFARI medication questionnaire. Of these individuals, 2 deletion carriers and no duplication carriers were currently taking antiepileptic drugs at the time of the VEP session.

Participants were not given an ophthalmological exam at the time of the VEP session due to budget and time constraints, but information related to eye surgeries, cataract, coloboma, glaucoma, and corrected vision was available on the SFARI developmental and medical history form for 17/19 deletion carriers and 8/9 duplication carriers. A total of 7 deletion carriers and 3 duplication carriers were reported to have corrected vision but no other uncorrected visual problems.

### Visual evoked potential recordings

Pattern VEPs were recorded as described previously [4]. Participants sat 60 cm from a Tobii Eye Tracker monitor (Tobii Technology, Sweden). Phase reversing 99 % contrast black and white checkerboard patterns were presented on the monitor using ePrime software (Psychology Software Tools Inc., Pittsburgh, PA, USA). The diagonal check size was 0.5 cycles/degree (60’), and the reversal rate was once every 500 ms. Approximately 50–100 trials were presented to each participant, and VEP sessions lasted no longer than several minutes. Stimulus presentation was experimenter-driven based on verification of binocular fixation on the monitor.

Continuous electroencephalogram (EEG) was recorded from a 128-channel HydroCel Geodesic Sensor Net (Electrical Geodesics Inc., Eugene, OR, USA), referenced to Cz, amplified with a NetAmps 300 amplifier, and digitized at 500 Hz. Data were analyzed offline using NetStation 4.5.4 software. The signal was filtered (0.3–30 Hz), segmented into 300 ms post-stimulus recording periods, and baseline-corrected against the mean voltage during the 100 ms pre-stimulus period. A 34-ms offset that was consistent for all participants was accounted for at this point in the analysis (16 ms between the stimulus trigger and appearance on the monitor and 18 ms due to anti-aliasing filters within the Electrical Geodesics Inc. NetAmps 300 amplifier).

An automated artifact detection tool flagged channels with excessive voltage change (>150 uV), and bad channels were replaced using spherical spline interpolation. Trials were removed if more than 10 % of channels were flagged or if the electrode of interest Oz was marked bad by artifact detection. Trials containing eye blinks, eye movements, or excessive noise or drift based on visual inspection were also removed from subsequent analysis. Average waveforms for each participant were generated and re-referenced to the average reference. Participants with fewer than 25 remaining trials were excluded from the dataset. There was no significant difference in the number of good trials between groups (Table 2).

**Table 2:** Quantification of VEP components and comparison between groups

	Control	Deletion	Duplication	Group comparisons	Pairwise comparisons
Good trials	63 (49)	60 (39)	45 (47)	H(2): 0.22, *p* = 0.894	–
Latency measurements (ms)					
N1 latency	60 (15)	46 (30)	46 (16)	**H(2): 6.85,** ***p*** **= 0.033**	*Ctr/Del: p = 0.053*
					*Ctr/Dup: p = 0.099*
					*Del/Dup: p = 1.000*
P1 latency	94 (9)	92 (12)	90 (7)	H(2): 1.72, *p* = 0.422	–
N2 latency	164 (71)	164 (44)	152 (81)	H(2): 0.13, *p* = 0.937	–
P1-N2 time	72.0 (66.0)	72.0 (50.0)	72.0 (76.0)	H(2): 0.37, *p* = 0.858	–
Amplitude measurements (uV)					
N1 amp	−4.5 (5.1)	−1.2 (5.4)	−3.4 (4.3)	H(2): 3.97, *p* = 0.137	–
P1 amp	10.4 (8.1)	15.7 (11.2)	5.4 (9.5)	**H(2): 11.92,** ***p*** **= 0.003**	*Ctr/Del: p = 0.088*
					*Ctr/Dup: p = 0.628*
					***Del/Dup: p = 0.003***
N2 amp	−0.94 (11.5)	−7.45 (7.3)	−3.19 (7.2)	H(2): 1.55, *p* = 0.460	–
N1-P1 amp	15.0 (17.5)	16.9 (6.1)	8.3 (11.1)	**H(2): 6.81,** ***p*** **= 0.033**	*Ctr/Del: p = 1.000*
					*Ctr/Dup: p = 0.226*
					***Del/Dup: p = 0.028***
P1-N2 amp	12.9 (17.6)	19.7 (16.4)	8.1 (10.3)	**H(2): 10.48,** ***p*** **= 0.005**	*Ctr/Del: p = 0.207*
					*Ctr/Dup: p = 0.455*
					***Del/Dup: p = 0.005***

Median values (interquartile range) are reported. Amp: amplitude. Pairwise comparisons with adjusted *p* values were performed if the overall group comparison was significant (*p* < 0.05). Significant results are in bold

### Visual evoked potential analysis

We analyzed activity over the midline occipital electrode Oz. The N1, P1, and N2 components were identified for each participant’s average waveform, which are equivalent to the N75, P100, and N135 [12] or the C1, P1, and N1 [15], respectively. The P1 was identified visually as the first prominent positive inflection point closest to 100 ms and occurred within 56–132 ms post-stimulus onset for all participants. The N1 was the negative inflection point immediately preceding the P1 and occurred within 0–70 ms post-stimulus onset for all participants. The N2 was the first negative inflection point following the P1 (or the last peak in a series of multiple peaks, as described previously [4]) and occurred within 108–266 ms post-stimulus onset in all participants. The absolute amplitude and latency of each component, as well as the relative amplitudes and times between components, were quantified for each individual’s average waveform.

### Statistical analysis

Statistical analysis was performed with IBM SPSS Statistics v.21 software. All data were analyzed blind to group and based on biological replicates (each data point is from one participant). Nonparametric tests were used due to small sample sizes and non-normal distribution of data. Results were considered significant if *p* < 0.05. The three participant groups (control, deletion, and duplication) were compared using a Kruskal-Wallis test, reported as H(df) where H is the test statistic and df is the degrees of freedom. If significant, the Kruskal-Wallis test was followed by paired comparisons with adjusted *p* values. Spearman correlations were used to compare the relationships between continuous variables.

## Results

**Fig. 1:**
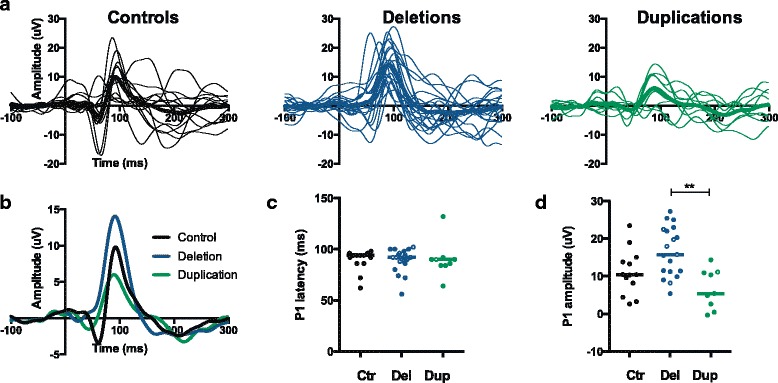
VEP waveforms in children with 16p11.2 deletions and duplications. **a** Averaged waveforms are displayed for each individual (*thin traces*) and each group (*thick traces*) for controls (*black*), deletion carriers (*blue*), and duplication carriers (*green*). The x-axis plots time in milliseconds, where −100 to 0 is the baseline period, 0 is the onset of the stimulus, and 0 to 300 is the response to the stimulus. The y-axis plots amplitude in microvolts. **b** Group average responses are displayed in an overlapping fashion to facilitate comparison between groups. **c** P1 latency is quantified for each individual. *Open circles* indicate individuals with a positive diagnosis for ASD. The *line* indicates the median for each group. **d** P1 amplitude is quantified for each individual. *Open circles* indicate individuals with a positive diagnosis for ASD. The *line* indicates the median for each group. **p < 0.01

We obtained usable data from 19 out of the 19 deletion carriers, 9 out of the 12 duplication carriers, and 13 out of the 15 control children tested (aged 3–14 years) (Table 1). Individual and group average waveforms are displayed in Fig. 1. Control waveforms were consistent with those reported in the literature, containing clearly identifiable N1, P1, and N2 components [12]. Deletion carriers had VEP waveforms that were generally larger and had a sharp return after peak response, while duplication carriers had VEPs that were smaller in amplitude and more heterogeneous in morphology (Fig. 1).

When comparing the three groups, no differences in latencies of any component were apparent, with the exception of a difference in N1 latency that did not remain significant for any paired comparisons (Table 2). Instead, there was a consistent effect on the amplitude of the principal positive component (P1), including absolute P1 amplitude and relative N1-P1 and P1-N2 amplitudes. This effect was due to a tendency towards large amplitudes in deletion carriers and small amplitudes in duplication carriers (Table 2, Fig. 1).

Consistent with the literature on the maturation of the pattern-reversal VEP [7–11], we observed no association between age and quantification of the VEP in any of our groups (Table 3). A relationship between IQ and pattern-reversal VEP amplitude has been reported in older children and adults [16]. As part of the Simons VIP project, IQ was assessed for most 16p individuals (17/19 deletion carriers and 7/9 duplication carriers). The deletion and duplication groups were well-matched for IQ (deletion: median = 81, mean = 82, range = 60–116; duplication: median = 81, mean = 83, range = 72–114). IQ was not assessed in our control group due to the design of the study, and we did not have enough duplication carriers with IQ measurements to perform a meaningful correlation. We failed to observe any correlation between IQ and any element of the VEP in our 16p deletion sample (Table 3).

**Table 3:** Correlations with age and IQ

	Correlation with age						Correlation with IQ	
Measurement	Control		Deletion		Duplication		Deletion	
	*r*	*P*	*r*	*P*	*r*	*P*	*r*	*P*
N1 latency	−0.02	0.957	−0.11	0.665	−0.66	0.052	−0.15	0.567
P1 latency	0.13	0.667	0.17	0.483	0.00	0.991	−0.35	0.163
N2 latency	0.37	0.220	0.02	0.950	0.03	0.932	0.07	0.793
P1-N2 time	0.41	0.162	−0.07	0.782	0.17	0.672	0.18	0.500
N1 amp	−0.10	0.747	−0.10	0.673	0.02	0.966	−0.19	0.455
P1 amp	−0.07	0.830	−0.18	0.468	−0.03	0.932	−0.09	0.729
N2 amp	0.08	0.788	0.22	0.359	−0.41	0.271	0.20	0.444
N1-P1 amp	0.09	0.774	0.15	0.552	−0.13	0.730	0.05	0.844
P1-N2 amp	−0.11	0.719	−0.14	0.569	0.09	0.813	−0.06	0.829

Amplitude (amp) values are in uV and latency (lat) or time values are in milliseconds. Spearman correlations were performed and the correlation coefficient (*r*) is reported. Correlation with IQ was only performed within the deletion group

Head size is known to be differentially affected by 16p11.2 copy number variation [1] and this could impact VEP latency and/or amplitude [17, 18]. Head circumference was measured at the beginning of the session in order to select an appropriate size net for each participant. While there was no statistically significant difference in head size between groups (H(2):4.06, *p* = 0.131), children carrying 16p duplications did tend to have smaller heads (TD: median = 52.0, mean = 52.4, range = 48.5–55.5; deletion: median = 51.5, mean = 52.4, range = 48.0–58.0; duplication: median = 49.8, mean = 50.1, range = 46.0-53.0; unit = cm). There was also no correlation within any group between head circumference and any VEP measurement, including N1-P1 amplitude (TD: *r* = 0.47, *p* = 0.127; deletion: *r* = 0.17, *p* = 0.494; duplication: *r* = 0.26; *p* = 0.108). Taken together, differences in head size are unlikely to explain the group differences in N1-P1 amplitude.

16p11.2 CNV syndrome increases the risk for autism spectrum disorder (ASD), particularly for those carrying deletions [19, 20]. Only a small number of individuals in our dataset had a confirmed diagnosis of ASD (*n* = 5 deletion carriers, *n* = 1 duplication carrier), and we designated these individuals as open circles in the scatter plots in Fig. 1c, d in order to visualize any trends. All individuals with ASD had P1 latencies close to the median value for their respective group (Fig. 1c). Individuals with ASD tended to have P1 amplitudes above the median value for their respective group. This result indicates that individuals carrying 16p deletions and a diagnosis of ASD may have larger VEP amplitudes, but future analysis of larger datasets is necessary.

## Discussion

The differing effect of 16p11.2 deletion and duplication on VEP P1 amplitude is intriguing and may offer insight into the neural differences between the two genetic conditions. It is known that deletion and duplication have opposite effects on head circumference (macrocephaly and microcephaly, respectively) [1], brain volume [13], and white matter microstructure [21]. Little is understood about how brain size and volume may affect VEP amplitude measured from the scalp, but this could be a contributing factor. It must also be considered that reduced cortical thickness has been reported in both deletion and duplication carriers [22], a finding that may be particularly relevant for a cortical recording method like VEP.

A reduction in P1 amplitude of the VEP has been found in psychoses [23–25], and 16p11.2 CNVs are associated with schizophrenia and bipolar disorder [20, 26]. This potential association could be investigated further in an older cohort of individuals with 16p11.2 CNVs.

A similar increase in VEP P1 amplitude has been found in mice carrying deletion of 16p11.2 (S. Cooke and M. Bear, personal communication). If this holds true, this parallel result could validate the translatable relevance of the mouse model to the human condition and will ultimately help us to better understand the synaptic and circuit mechanisms underlying cortical dysfunction in 16p CNV syndrome. Some recent in-depth analysis of cortical organization has been done in mice carrying 16p deletions. Specifically, these mice display fewer upper layer pyramidal cells and increased layer 6 corticothalamic projection neurons due to ERK dysregulation [14]. Such changes in cortical microcircuitry could contribute to the changes in VEP amplitude observed in both mice and humans.

Interestingly, auditory responses measured using magnoencephalography were reported to be delayed in children with 16p deletions but not duplications, and there was no contribution of age or IQ to this result [5]. The representative traces displayed in the first figure of that paper appear to show increased signal amplitude in deletions and decreased amplitude in duplications; however, quantification of amplitude was not reported so this may not be a significant finding. Here, we used EEG to assess visual processing in our study and found differences in amplitude and not latency. It is unclear if our findings differ due to the sensory system that was being assessed or the method used.

### Limitations of our study

One potential limitation of our study is the broad age range of our sample that spans childhood. However, the VEP is mature by 2 years of age [7–11], which is well below the age of our youngest participant, and we saw no effect of age on any VEP component within any group. Another limitation is the small sample size and variability of phenotype and comorbid conditions, including seizure history, medication use, and ASD diagnosis. This is a challenge inherent in studying this population and means that any differences that emerge from group comparison are then potentially specific to the genetic condition. Cognitive ability is one phenotypic variable that we were not able to adequately address in this study because IQ was not assessed in the typically developing group due to the nature of the study design. IQ did not differ between the 16p deletion and duplication groups, however, and because there was no correlation between IQ and VEP amplitude in the deletion group, IQ is not likely to explain the differences in P1 amplitude in our dataset.

Finally, because visual information must first travel through the retina and thalamus before reaching the cortex where the VEP is recorded, we cannot exclude that ophthalmological abnormalities could contribute to any group differences we see. However, no participant was reported to have had eye surgery, cataract, coloboma, glaucoma, or any other uncorrected visual problems. In addition, the latency of the components was unaffected, suggesting that the arrival of visual information from the periphery to the cortex was intact.

## Conclusions

Our study has shown that it is possible to successfully record VEP in children with 16p11.2 CNV disorder who present with a range of intellectual, behavioral, and physical disabilities. We have shown that VEP responses are robust, easily quantifiable, and unaffected by age, head circumference, or IQ. Furthermore, the amplitude of the principal P1 component is enhanced in deletion carriers and diminished in duplication carriers, providing a biomarker to further probe cortical processing in the two conditions.

## Abbreviations

ASD, autism spectrum disorder; BCH, Boston Children’s Hospital; CNV, copy number variation; EEG, electroencephalogram; IQ, intelligence quotient; SFARI, Simons Foundation Autism Research Initiative; SVIP, Simons Variation in Individuals Project; VEP, visual evoked potential
